# A *MUCINs* expression signature impacts overall survival in patients with clear cell renal cell carcinoma

**DOI:** 10.1002/cam4.4128

**Published:** 2021-07-29

**Authors:** Hui Meng, Xuewen Jiang, Huangwei Huang, Neng Shen, Changsheng Guo, Chunxiao Yu, Gang Yin, Yu Wang

**Affiliations:** ^1^ Department of Urology Qilu Hospital Jinan Shandong China; ^2^ Cheeloo College of Medicine Shandong University Jinan Shandong China; ^3^ Department of Surgery Taian TSCM hospital Taian Shandong China; ^4^ Department of Urology Liaoning Hospital of Traditional Chinese Medicine Dezhou Shandong China; ^5^ Department of Urology Central Hospital of Zaozhuang Mining Group Shandong China; ^6^ Center for Reproductive Medicine Department of Obstetrics and Gynecology Qilu Hospital Jinan Shandong China

**Keywords:** ccRCC, MUCIN, overall survival, prognosis, unsupervised hierarchical clustering

## Abstract

**Background:**

Kidney cancer, especially clear cell renal cell carcinoma (ccRCC), is one of the most common cancers in the urinary system. Previous studies suggested that certain members of *MUCIN*s could serve as independent predictors for the survival of ccRCC patients. None of them, however, is robust enough to predict prognosis accurately.

**Objective:**

To analyze the correlation of *MUCINs* alterations and their expression levels with the prognosis of ccRCC patients and develop a prognosis‐related predictor.

**Methods:**

We applied whole‐exome sequencing in samples from 22 Chinese ccRCC patients to identify genetic alterations in *MUCIN* genes and analyzed their genetic alterations, expression, and correlation with survival using the TCGA, GSE73731, and GSE29069 datasets.

**Result:**

Genetic alternations in *MUCINs* were identified in 91% and 51% of ccRCC patients in our cohort and the TCGA database, respectively. No correlation with survival was found for the genetic alterations. Using unsupervised clustering analysis of gene expression, we identified two major clusters of *MUCIN* expression patterns. Cluster 1 was characterized by a global overexpression of *MUC1*, *MUC12*, *MUC13*, *MUC16*, and *OVGP1*; and cluster 2 was characterized by a global overexpression of *MUC4*, *MUC5B*, *MUC6*, *MUC20*, *EMCN*, and *MCAM*. Patients with cluster 1 expression pattern had significantly shorter overall survival time and worse clinical features, including higher tumor grades and metastasis. Meanwhile, they had a higher level of mutation counts and more infiltrated immune cells, but lower enrichment in angiogenesis signature genes. A five‐*MUCINs* expression signature was constructed from cluster 1, and notably, it was demonstrated to be associated with shorter overall survival. A similar worse clinical feature, lower angiogenesis but the more immune signature, was identified in samples presented with signature 1. In the validation data set GSE29069, patients with signature 1 were also associated with a trend of poor survival outcomes.

**Conclusion:**

We established a five‐*MUCINs* expression signature as a new prognostic marker for ccRCC. The distinct tumor microenvironment feature between the two signatures may further affect ccRCC patients’ clinical management.

## Graphical abstract

We identified a five‐MUCINs expression signature associated with the prognosis for ccRCC patients, which provided a new prognostic marker for ccRCC patients. The distinct tumor microenvironment feature between the two signatures may further affect ccRCC patients’ management.

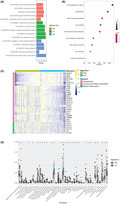

## 1 INTRODUCTION

Renal carcinoma is a malignant neoplasm originating from the urinary tubular epithelial of the renal parenchyma.^1^ Worldwide, the incidence and death of renal carcinoma are continuously rising, with a predicted incidence of 403,262 cases and 175,098 deaths in 2018.^2^ In China, approximately 68,300 new cases were diagnosed and 25,600 deaths attributed to renal carcinoma^3^ The five‐year survival rate for patients with metastatic ccRCC is less than 10%.^4^ Even for those with localized tumors, the risk for recurrence or metastatic following complete resection is as high as 40%.^5^

Tumor grade and histological features were found to be correlated with prognosis in ccRCC patients.^6^ Nevertheless, intratumor and intertumoral heterogeneity of the same patient is conspicuous, which complicates the prediction accuracy by pathology.^7^, ^8^ Molecularly classification of ccRCC includes the analysis of single‐nucleotide polymorphism, somatic mutations, DNA methylation, and gene expression.^9^, ^10^ Integrative analysis of genetic mutation and clinical information demonstrated that several genetic changes, such as *BAP1* mutation, were associated with poor clinical outcomes in ccRCC patients as independent predictors.^11^ However, these prognostic markers are not robust enough for clinical practice.

MUCINs are epithelial cells‐expressed large O‐glycoproteins, and their overexpression and glycosylation in malignancies are found to facilitate oncogenic processes.^12^ Previous studies have found that the expression levels of *MUCINs*, including *MUC1*, *MUC4*, *MUC12*, *MUC13*, *MCAM*, and *LAMA4* were correlated with poor survival of ccRCC patients, and/or promotion of tumor cell proliferation in vitro.^13^, ^14^, ^15^, ^16^, ^17^, ^18^ *MUCIN*s could be classified into secreted, membrane‐bounded, and atypical types according to their structure and localization; however, the specific biological functions of different types in the prognosis of ccRCC are undefined. Previous studies mainly focused on the individual member of *MUCIN*’s correlation with prognosis in ccRCC and a comprehensive evaluation of the *MUCIN* family members in ccRCC is still lacking.

In this study, we analyzed the correlation of patients’ survival with genetic alteration and mRNA expression profile of *MUCIN*s in ccRCC patients. Subsequently, we developed and validated a combined *MUCIN*s expression‐based signature for predicting the prognosis of ccRCC, which is worth being validated prospectively and utilized in further clinical practice.

## 2 METHODS

### 2.1 Data resource and study design

Whole‐exome sequencing was performed for tumor tissues from 22 ccRCC patients from Qilu hospital and genomic alternation profiles were identified in 20 *MUCIN*s genes.

Meanwhile, publicly available *MUCIN*s genomic alternation data and corresponding clinical feature information (including overall survival, neoplasm disease grade, tumor stage, and metastasis stage) of ccRCC were downloaded from cBioPortal for Cancer Genomics database (https://www.cbioportal.org/), which contained 538 ccRCC samples. Transcriptome data for 100 cases of normal kidney tissue were downloaded from Genome Tissue Expression (GTEX).

Then, data sets GSE73731 and GSE29069 were downloaded from Gene Expression Omnibus (GEO) database (http://www.ncbi.nml.nih.gov/geo/). The data set GSE73731 included *MUCIN*s genomic alternation data of 265 ccRCC samples, which was used to verify the correlation of *MUCIN*s expression with prognosis. The data set GSE29069 containing 39 ccRCC samples and survival data were applied for validation of the findings for prognosis.

### 2.2 Screening of differentially expressed *MUCINS*

In the preprocessing of the raw data, *MUCIN*s with low‐quality data were excluded. GEPIA server was applied to analyze the *MUCIN*s expression profile in ccRCC compared with normal tissue controls. *P* <= 0.05 and logarithmic fold changes >=0.3 were considered statistically significant.^19^ GEPIA is available at http://gepia.cancer‐pku.cn/. All plotting features in GEPIA are developed using R (version 3.3.2).

### 2.3 Clustering analysis of CCRCC patients

The Pearson correlation coefficients (*r*‐values) and *p*‐values for each combination of *MUCIN* genes were calculated using the Hmisc package in R studio. Principal component analysis (PCA) and unsupervised hierarchical clustering were performed using the FactoMineR package in R studio. The *MUCIN* members with no expression in 20% samples or more were excluded.

### 2.4 Survival analysis

Kaplan–Meier survival curves and Cox survival analysis were generated by the “survival” package and “survminer” package in R. The Cox proportional hazard ratio and the 95% confidence interval were included in the survival plot.

### 2.5 Gene enrichment analysis

Gene ontology (GO) and Kyoto Encyclopedia of Genes and Genomes (KEGG) pathway enrichment analysis were conducted using the “ClusterProfiler” package in R. We used “adjusted *p* < 0.05” as the significant criteria and selected the GO terms and KEGG pathways with the highest statistical significance.

### 2.6 XCELL and GENE set variation analysis

We estimated the abundance of different immune and stromal cell types in each ccRCC case using xCell, an analysis of the cellular heterogeneity landscape through tumors gene profiles, which includes 64 different immune and stromal cell types.^20^ xCell is available at http://xCell.ucsf.edu/.

### 2.7 Statistical analysis

Student's *t* test and non‐parametric test (Chi‐square analysis of variance and Wilcoxon Rank‐sum test) were used for the comparison of molecular differences and clinical features between two groups using R studio (https://rstudio.com/). A log‐rank test was used to evaluate the survival curves between different groups. And *p*‐value < 0.05 was considered statistically significant.

## 3 RESULTS

### 3.1 Genetic alteration in MUCIN genes

**FIGURE 1:**
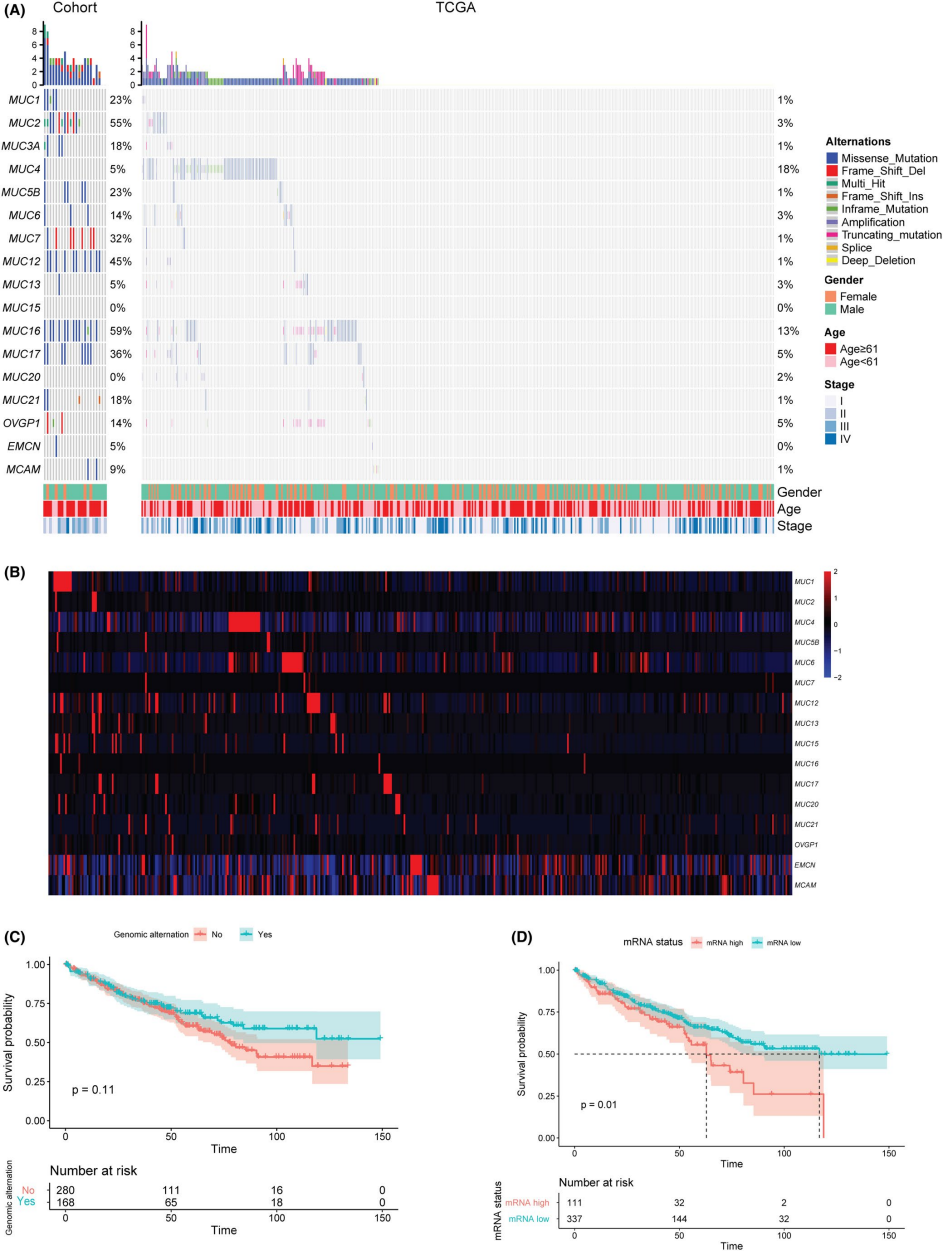
*MUCIN*s genomic alternation and survival in the ccRCC cohort. (A) *MUCIN*s genomic alternation profiles (gene amplification, deletion, mutations) in the ccRCC patients of the Chinese cohort (n = 22) and TCGA dataset (n = 448). (B) The heatmap of the *MUCIN*s mRNA expression for each patient in the TCGA dataset (n = 446). (C) Kaplan–Meier curves of clusters with and without genomic alternation (*p* = 0.11). (D) Kaplan–Meier curves of clusters with mRNA high and low expression (*p* = 0.01)

In our ccRCC cohort, 91% (20/22) cases were identified as having at least one alternation in *MUCINs*, with the most prevalence in *MUC16*, *MUC2*, and *MUC12* (59%, 56%, and 45%, respectively). Meanwhile, only 52% (235/448) of ccRCC cases had at least one genetic alternation in *MUCIN*s in the TCGA database, but the majority had alternations in *MUCIN*s mRNA expression levels (Figure 1A,B). There was no significant difference in the overall survival between ccRCC patients with and without genomic alternations in *MUCINs* (*p *= 0.11, Figure 1C). However, patients with higher mRNA expression in *MUCINs* had significantly worse survival (*p *= 0.01, Figure 1D).

### 3.2 *MUCIN* expression pattern in CCRCC

**FIGURE 2:**
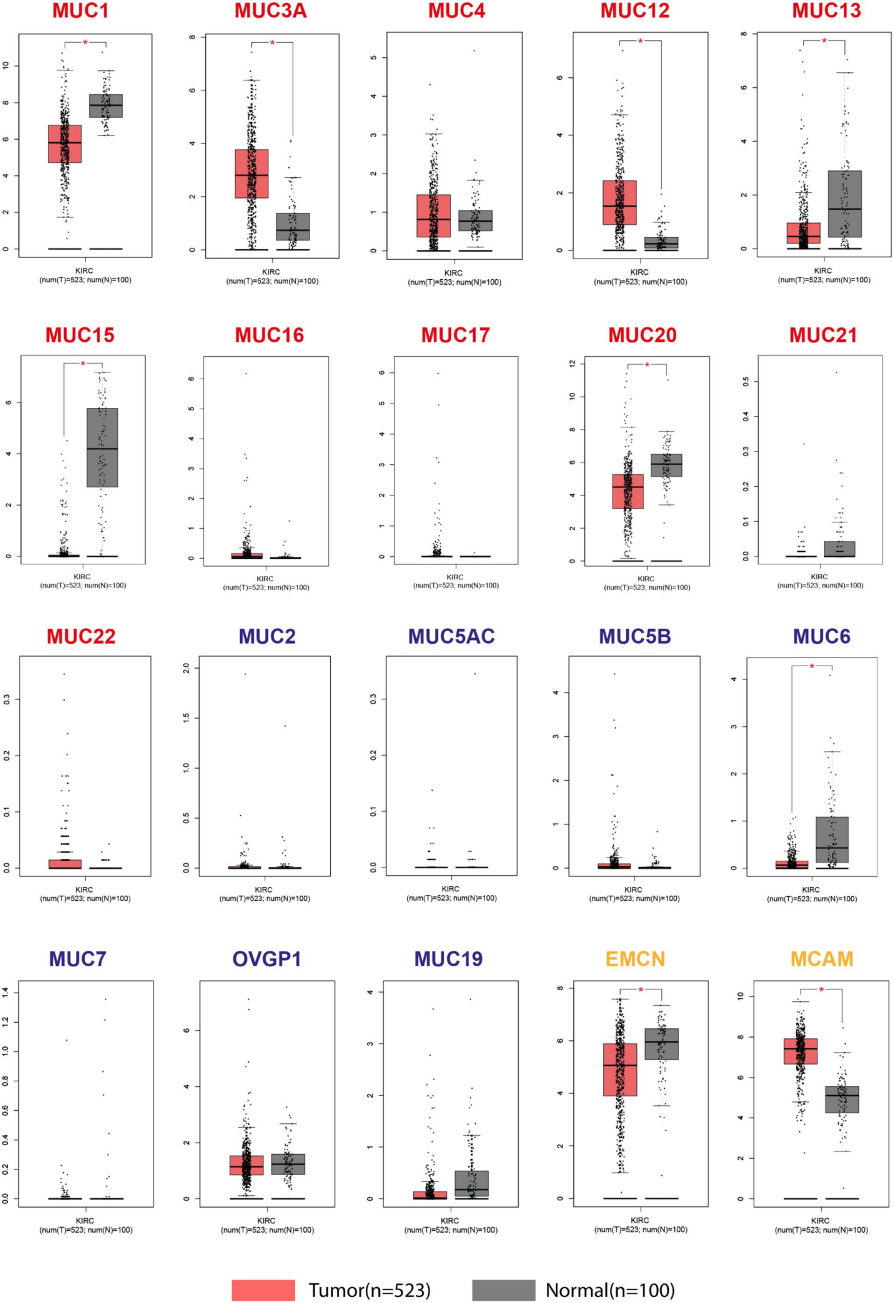
Relative mRNA expression levels in ccRCC and normal renal tissues. Membrane‐bound MUCINs (*MUC1*, *MUC3A*, *MUC4*, *MUC12*, *MUC13*, *MUC15*, *MUC16*, *MUC17*, *MUC20*, *MUC21*, and *MUC22*), secreted MUCINs (*MUC2*, *MUC5AC*, *MUC5B*, *MUC6*, *MUC7*, *OVGP1*, and *MUC19*) and atypical MUCINs (*EMCN* and *MCAM*) were labeled with red, blue, and orange titles, respectively. Statistical analyses were performed using unpaired t‐test (* indicates *p* < 0.05)

GEPIA web‐server was applied to assess the *MUCINs* expression level in ccRCC patients compared to the normal controls. Expression levels of 9 *MUCINs* were significantly altered in ccRCC patients (*p *< 0.05), including six membrane‐bound *MUCINs* (*MUC1*, *MUC3A*, *MUC12*, *MUC13*, *MUC15*, *and MUC20*), one secreted *MUCIN* (*MUC6*), and two atypical *MUCINs* (*EMCN* and *MCAM*) (Figure 2). Specifically, three membrane‐bound *MUCINs* (*MUC3A*, *MUC12*, and *MUC20*) and one atypical mucin (*MCAM*) were overexpressed in the tumor samples (*p* < 0.05). In contrast, three membrane‐bound *MUCIN*s (*MUC1*, *MUC13*, and *MUC15*), one atypical *MUCIN* (*EMCN*), and one secretion *MUCIN* (*MUC6*) were significantly downregulated in the tumor samples (*p* < 0.05).

### 3.3 The relationship between MUCIN expression and survival in CCRCC

**FIGURE 3:**
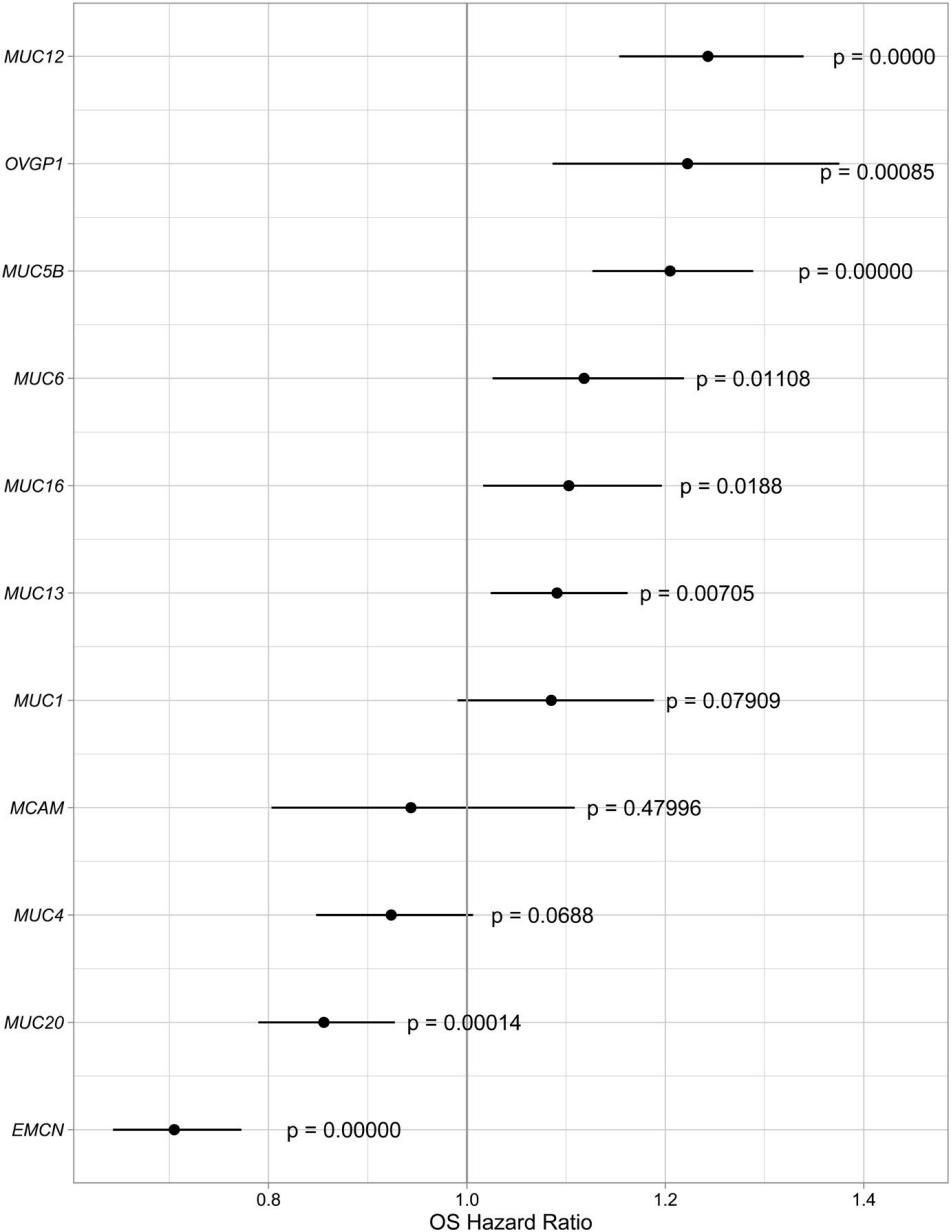
Hazard ratio of high and low expression levels of *MUCIN*s in the TCGA cohort

Excluding *MUCIN*s with low‐quality expression data, 11 *MUCIN*s were available for the subsequent analysis. Univariate Cox analysis was conducted to estimate the hazard ratio (HR) of *MUCIN*s expression in the survival of ccRCC patients. Significantly increased expression of *MUC12*, *OVGP1*, *MUC5B*, *MUC6*, *MUC16*, *MUC13* (HR = 1.09–1.24, *p *<0.05) and a trend of increased expression in *MUC1* (HR = 1.09, 95%CI = 0.99–1.19, *p *= 0.079) were identified in the patients with worse survival (Figure 3). Meanwhile, significantly lower expression of *MUC20* (HR = 0.86, 95%CI = 0.79–0.93, *p *= 0.0001) and *EMCN* (HR = 0.70, 95%CI = 0.64–0.77, *p* = 7.53E^−14^) were identified in the patients with worse survival (Figure 3).

### 3.4 Correlation of *MUCIN*S expression levels in CCRCC

**FIGURE 4:**
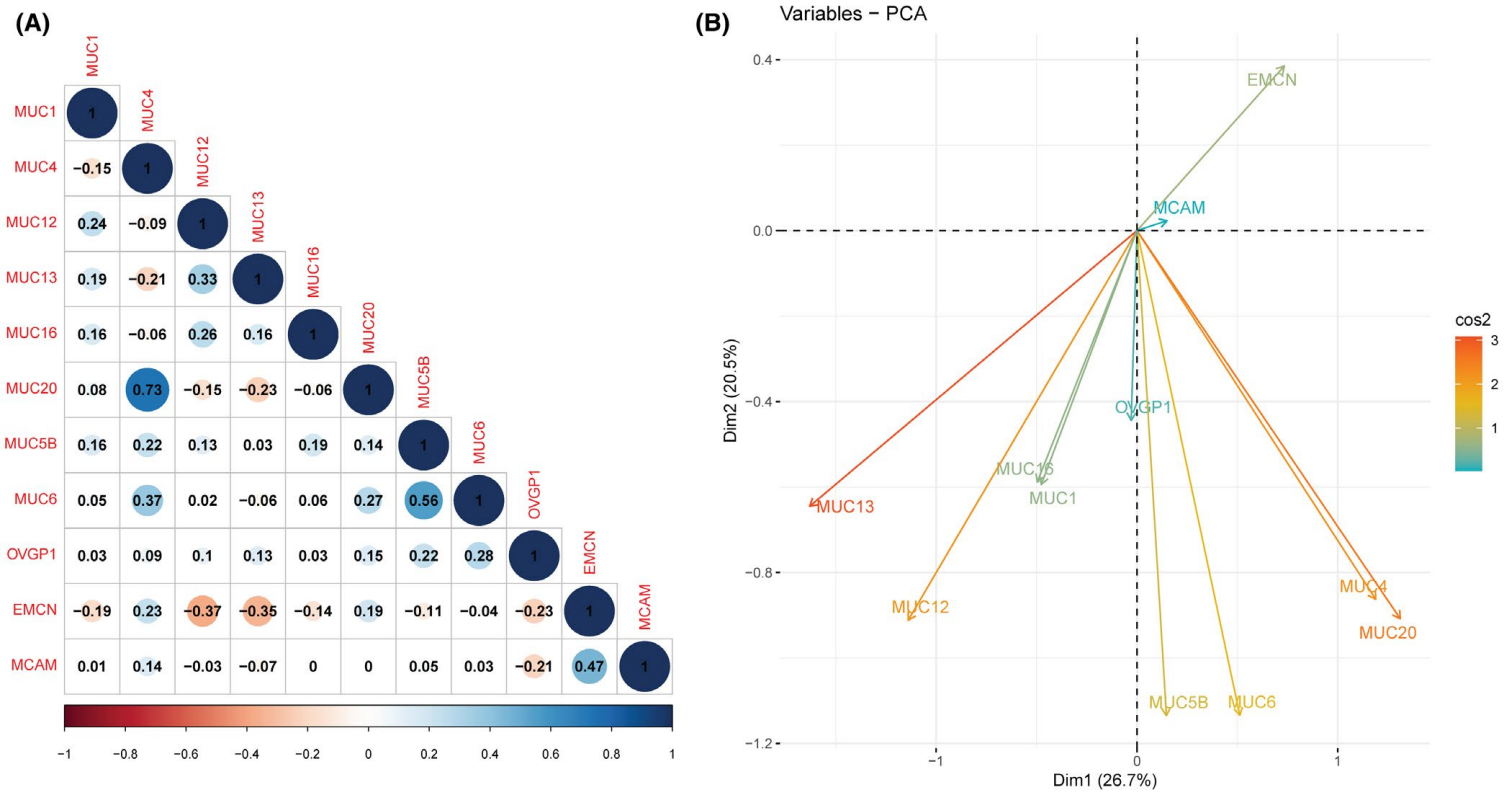
Correlation analysis of MUCINs mRNA levels in ccRCC cohort. (A) Pearson correlation between *MUCIN*s. (B) Principal component analysis (PCA) analysis of *MUCIN*s mRNA relative expression

To further understand the *MUCIN*s’ expression pattern in ccRCC patients, we calculated the Pearson correlation coefficient for each pair of *MUCIN*s and revealed a principal component analysis (PCA) for the 11 *MUCIN*s expression profile. There were 25 pairs of *MUCIN*s with a positive correlation (0.09 < *r* < 0.73, Table 1) and 12 with negative correlation (−0.37 < *r* < −0.09, Table 2) (*p *< 0.05, Figure 4A). We identified a strong coexistence between *MUC4* and *MUC20* (*r* = 0.73). Four membrane‐bound *MUCIN*s (*MUC1*, *MUC12*, *MUC13*, *MUC16*) and one secreted *MUCIN* (*OVGP1*) were negatively correlated with atypical *MUCINs* (*EMCN* and *MCAM*, −0.37 < *r* < −0.14). Meanwhile, *MUC4*, *MUC5B*, *MUC6*, and *MUC20* were grouped together (Figure 4Bb). Further validation analysis in the GSE73731 dataset showed 108 significant positive correlations and 31 significant negative correlations, as well as similar correlation expression patterns of *MUC1*, *MUC12*, *MUC13*, *MUC16*, *OVGP1*, and atypical *MUCINs (EMCN*, *MCAM)* (−0.40 < *r* < −0.13, Table S1, Figure S1).

**TABLE 1:** Positive correlation of Mucin gene expression in ccRCC‐TCGA

Mucins	Mucins	Pearson *r*	*p*
*MUC20*	*MUC4*	0.734007	0
*MUC6*	*MUC5B*	0.559456	0
*MCAM*	*EMCN*	0.468384	0
*MUC6*	*MUC4*	0.373256	0
*MUC13*	*MUC12*	0.334014	2.22E−15
*OVGP1*	*MUC6*	0.278651	5.59E−11
*MUC6*	*MUC20*	0.270565	2.06E−10
*MUC16*	*MUC12*	0.263322	6.41E−10
*MUC12*	*MUC1*	0.241057	1.69E−08
*EMCN*	*MUC4*	0.233499	4.79E−08
*OVGP1*	*MUC5B*	0.223327	1.84E−07
*MUC5B*	*MUC4*	0.222445	2.06E−07
*EMCN*	*MUC20*	0.193238	6.88E−06
*MUC5B*	*MUC16*	0.19	9.84E−06
*MUC13*	*MUC1*	0.187116	1.35E−05
*MUC16*	*MUC13*	0.161709	0.000175
*MUC16*	*MUC1*	0.161397	0.00018
*MUC5B*	*MUC1*	0.159313	0.000219
*OVGP1*	*MUC20*	0.145455	0.000748
*MCAM*	*MUC4*	0.141622	0.001032
*MUC5B*	*MUC20*	0.136833	0.001527
*OVGP1*	*MUC13*	0.132488	0.002155
*MUC5B*	*MUC12*	0.128844	0.002856
*OVGP1*	*MUC12*	0.100748	0.019881
*OVGP1*	*MUC4*	0.08808	0.041894

**TABLE 2:** Negative correlation of Mucin gene expression in ccRCC‐TCGA

Mucins	Mucins	Pearson *r*	*p*
*EMCN*	*MUC12*	−0.37185	0
*EMCN*	*MUC13*	−0.34702	0
*MUC20*	*MUC13*	−0.23436	4.26E−08
*EMCN*	*OVGP1*	−0.2261	1.28E−07
*MCAM*	*OVGP1*	−0.21355	6.33E−07
*MUC13*	*MUC4*	−0.21301	6.77E−07
*EMCN*	*MUC1*	−0.18511	1.67E−05
*MUC4*	*MUC1*	−0.15292	0.000391
*MUC20*	*MUC12*	−0.15079	0.000472
*EMCN*	*MUC16*	−0.14453	0.000809
*EMCN*	*MUC5B*	−0.10878	0.011892
*MUC12*	*MUC4*	−0.08943	0.038836

### 3.5 Clustering analysis in CCRCC

**FIGURE 5:**
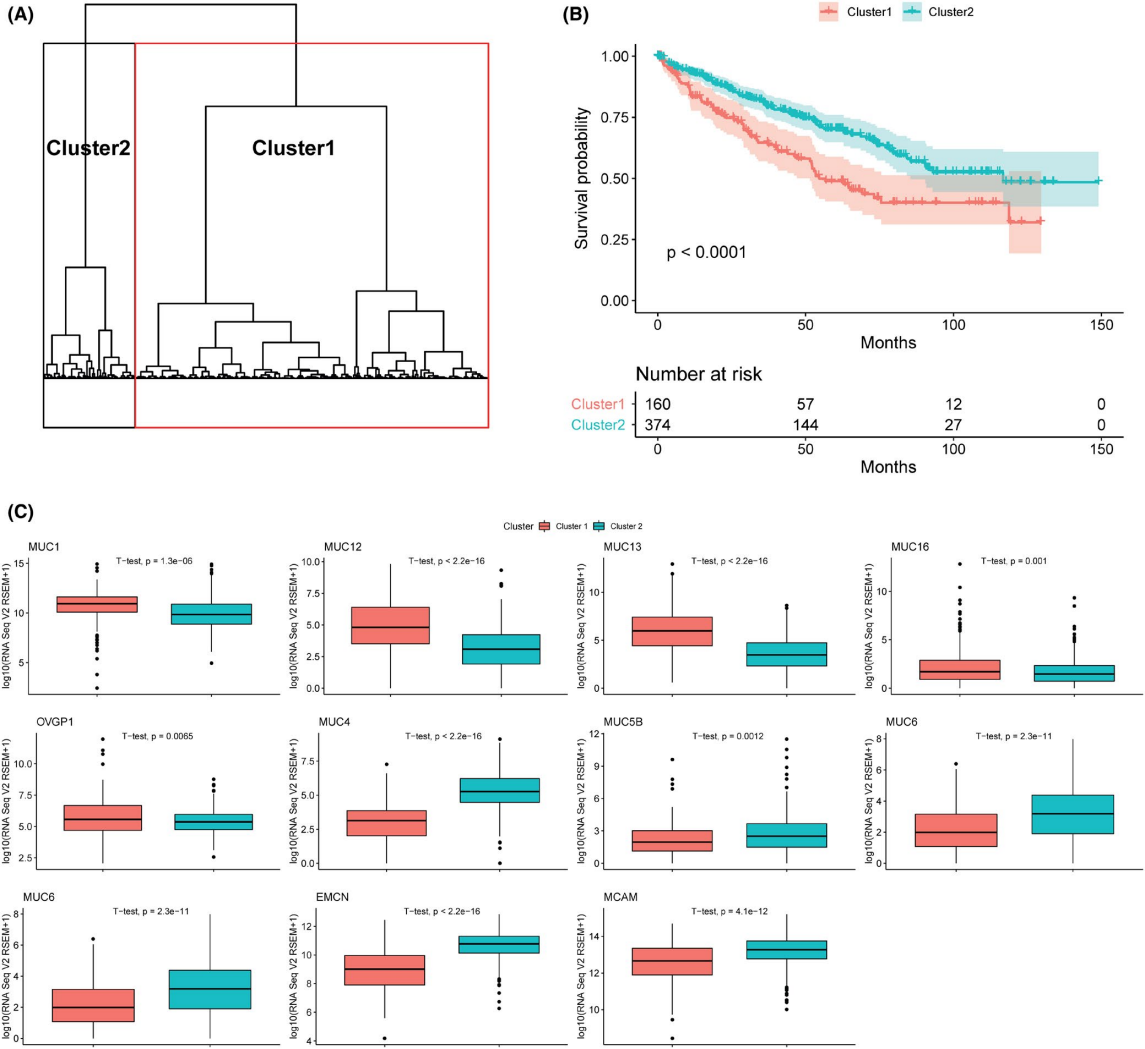
Clustering analysis in ccRCC cohort. (A) Unsupervised hierarchical clustering of *MUCIN*s profile. The dendrogram showed two clusters according to the expression pattern of mucins. (B) Survival analysis of two clusters (*p* < 0.0001). (C) Boxplot of *MUCIN*s expression in cluster 1 and cluster 2. Statistical analyses were performed using unpaired *t*‐test. *p*‐value < 0.05 was considered statistically significant

**FIGURE 6:**
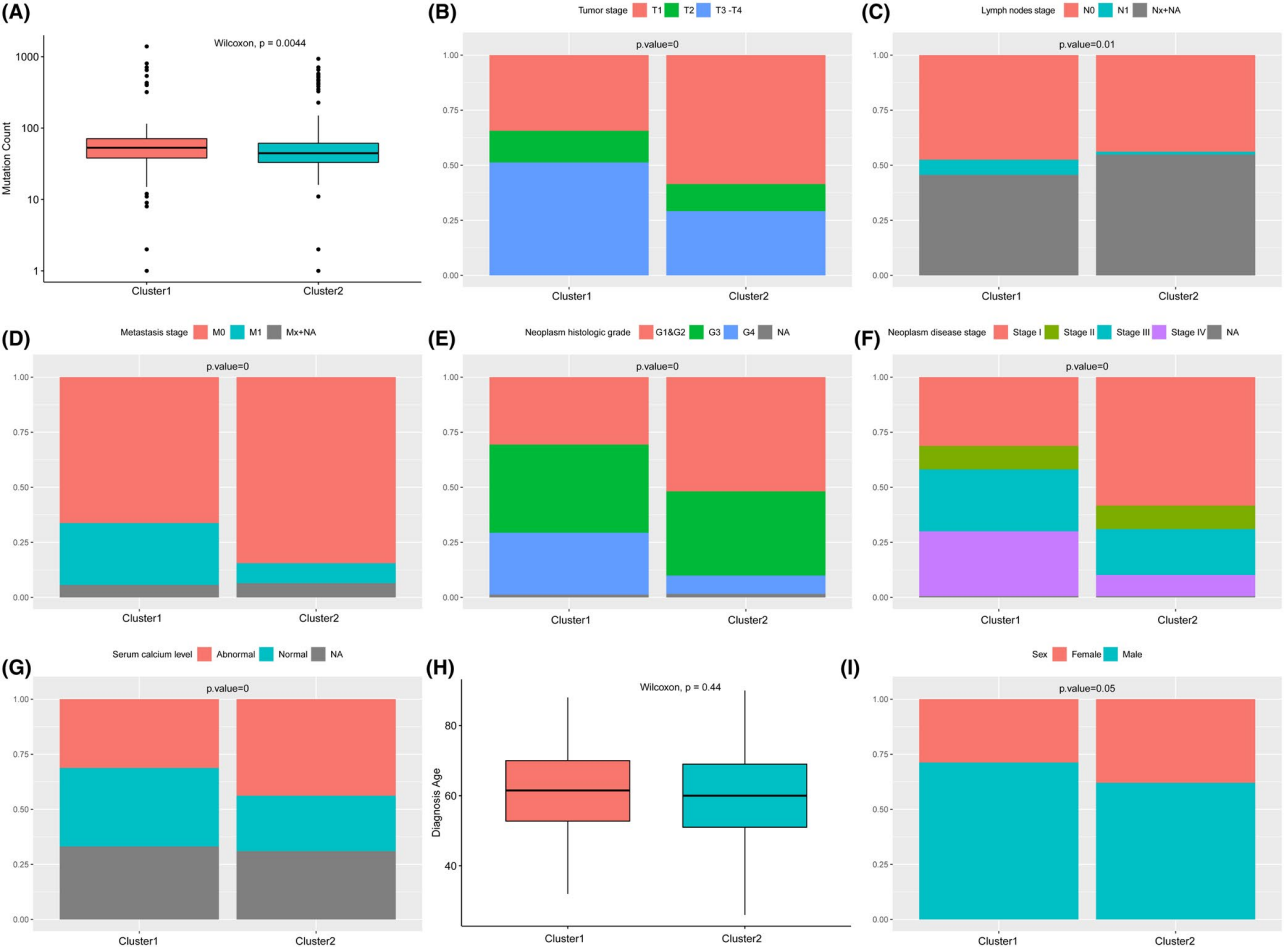
Clinical features analysis of the two clusters in the ccRCC cohort. (A) Mutation counts of clusters 1 and 2 in the TCGA cohort. Percentage‐staked bar plot of (B) tumor stage, (C) lymph nodes stage, (D) metastasis stage, (E) neoplasm histologic grade, (F) neoplasm disease stage, (G) serum calcium level in clusters 1 and 2. (H) Age at diagnosis and gender distribution in clusters 1 and 2

Unsupervised hierarchical clustering was applied according to samples’ *MUCIN* expression patterns and the ccRCC cases were classified into two distinct clusters (cluster 1 and cluster 2) (Figure 5A). There were 160 cases (29.96%) in cluster 1 and 374 (70.04%) in cluster 2. Notably, cluster 1 was significantly associated with worse survival (median OS: 54.57 vs. 116.75 months, *p *< 0.0001, Figure 5B). To further characterize each cluster, we performed an unpaired t‐test for two clusters on log‐transformed mRNA expression. There were five *MUCIN*s (*MUC1*, *MUC12*, *MUC13*, *MUC16*, and *OVGP1*) significantly overexpressed in cluster 1 (*p *< 0.05) and most of them were membrane‐bound *MUCIN*s except *OVGP1*. In contrast, six *MUCIN*s (*MUC4*, *MUC5B*, *MUC6*, *MUC20*, *EMCN*, and *MCAM*, *p *< 0.05) were overexpressed in cluster 2, which contained two membrane‐bound *MUCIN*s, two secreted *MUCIN*s, and all the atypical *MUCIN*s (Figure 5C). By investigating the clinical feature of these two clusters, a significant increase in the genetic mutation counts in patients from cluster 1 was noticed (median counts 53 vs. 44.5, *p* = 0.0044) (Figure 6A). Patients in cluster 1 were presented with later tumor stage, clinical stage, and higher neoplasm histological grade than those in cluster 2 (Figure 6B–G). There was no significant difference in the age at diagnosis (61 years old, Figure 6H) and gender between the two clusters (Figure 6I).

### 3.6 Tumor characteristics in different clusters

**FIGURE 7:**
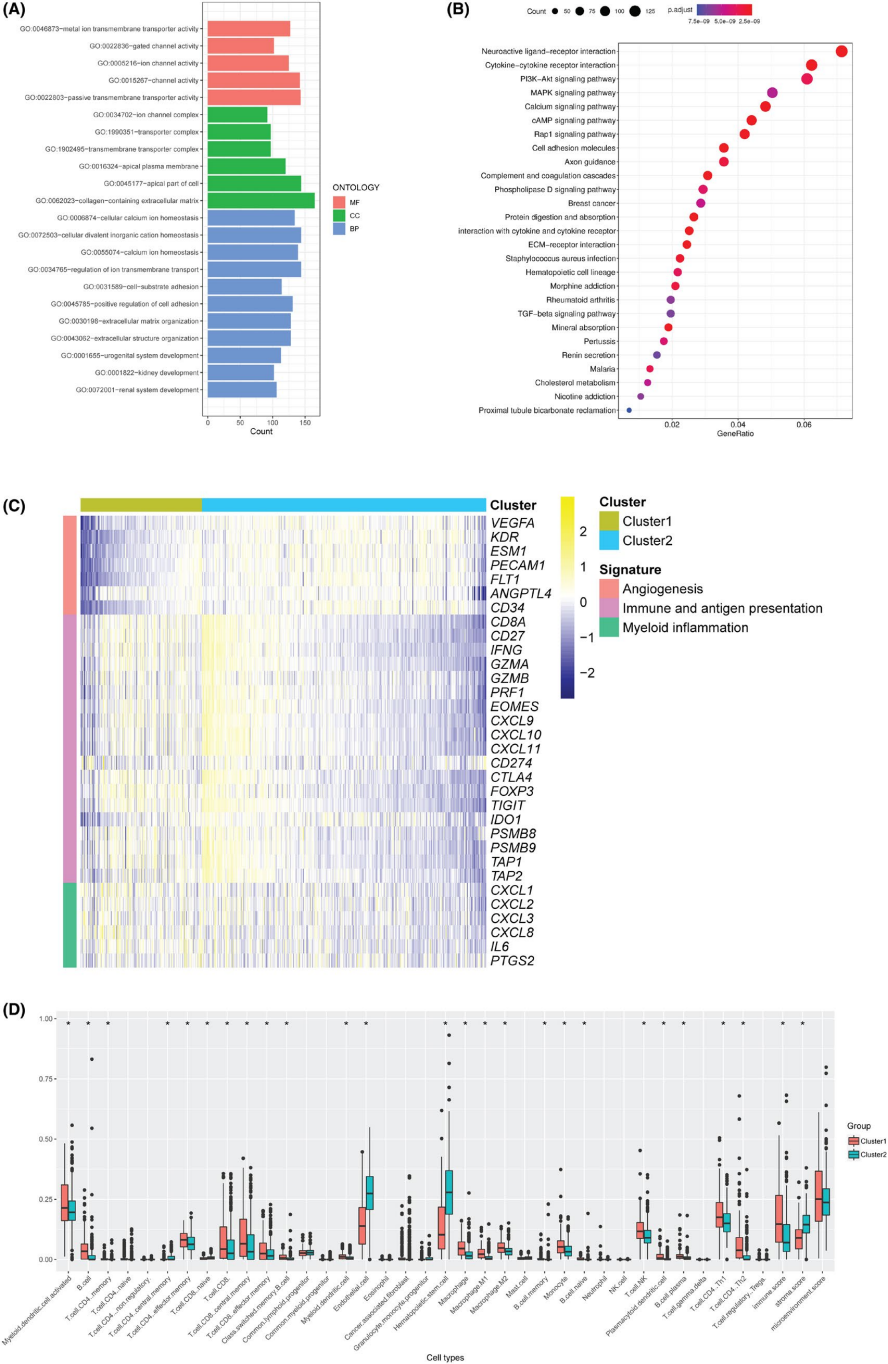
Relationship between clusters and most immune‐related biological pathways in the ccRCC cohort. (A) Gene ontology enrichment of DEGs. (B) KEGG enrichment of DEGs. (C) mRNA expressions heatmap of genes related to angiogenesis, immune, and antigen presentation, and myeloid inflammation. (D) Different distributions of cells are estimated by xCell in cluster 1 and cluster 2. BP: biological process; CC: cellular component; MF: molecular function. * represent *p* < 0.05

Compared with cluster 2, 1730 genes were upregulated and 1837 genes were downregulated in cluster 1. To further distinguish the biological features between the two clusters, we performed gene ontology (GO) and KEGG pathway enrichment analysis on the differentially expressed genes (DEGs). The following GO annotations of DEGs were found to be enriched (FDR < 0.05, Figure 7Aa): as for the molecular function, transporter and channel, extracellular matrix and receptor; as for the cellular component, extracellular matrix, apical region and transporter, and ion channel complex. Meanwhile, the significantly enriched DEGs were involved in renal system development, extracellular matrix and cell adhesion and calcium ion process. Similarly, KEGG analysis showed that the DEGs were mainly enriched in receptor interaction, extracellular matrix, cell adhesion, and calcium signaling pathway (Figure 7B). We also investigated the association between the two clusters and angiogenesis/immune biology with a predefined gene set. The heatmap indicated that genes related to angiogenesis had relatively lower expression level in cluster 1, but genes related to immune biology were upregulating instead (Figure 7C). By analyzing the composition of tumor microenvironment using xCell, it was revealed that cluster 1 was correlated with a higher immune cell level and microenvironment score, whereas cluster 2 had a higher stroma score (Figure 7D). As shown in Figure S2, the immune score was positively correlated with the expression levels of *MUC1* (*R* = 0.12, *p *= 0.0054), *MUC12* (*R* = 0.3, *p *< 0.001), *MUC13* (*R* = 0.26, *p *< 0.001), and *MUC16* (*R* = 0.23, *p *< 0.001). Meanwhile, CD4 positive effector memory T cells were positively correlated with *MUC1* (*R* = 0.1, *p* = 0.02), *MUC12* (*R* = 0.33, *p *< 0.001), *MUC13* (*R* = 0.27, *p *< 0.001), and *MUC16* (*R* = 0.13, *p* = 0.0038), whereas CD8 positive T cells were correlated with *MUC12 R* = 0.22, *p *< 0.001), *MUC13* (*R* = 0.22, *p *< 0.001), *MUC16* (*R* = 0.32, *p *< 0.001), and *OVGP1* (*R* = 0.11, *p* = 0.0095).

### 3.7 *MUCIN* signature and the prognosis of CCRCC patients

**FIGURE 8:**
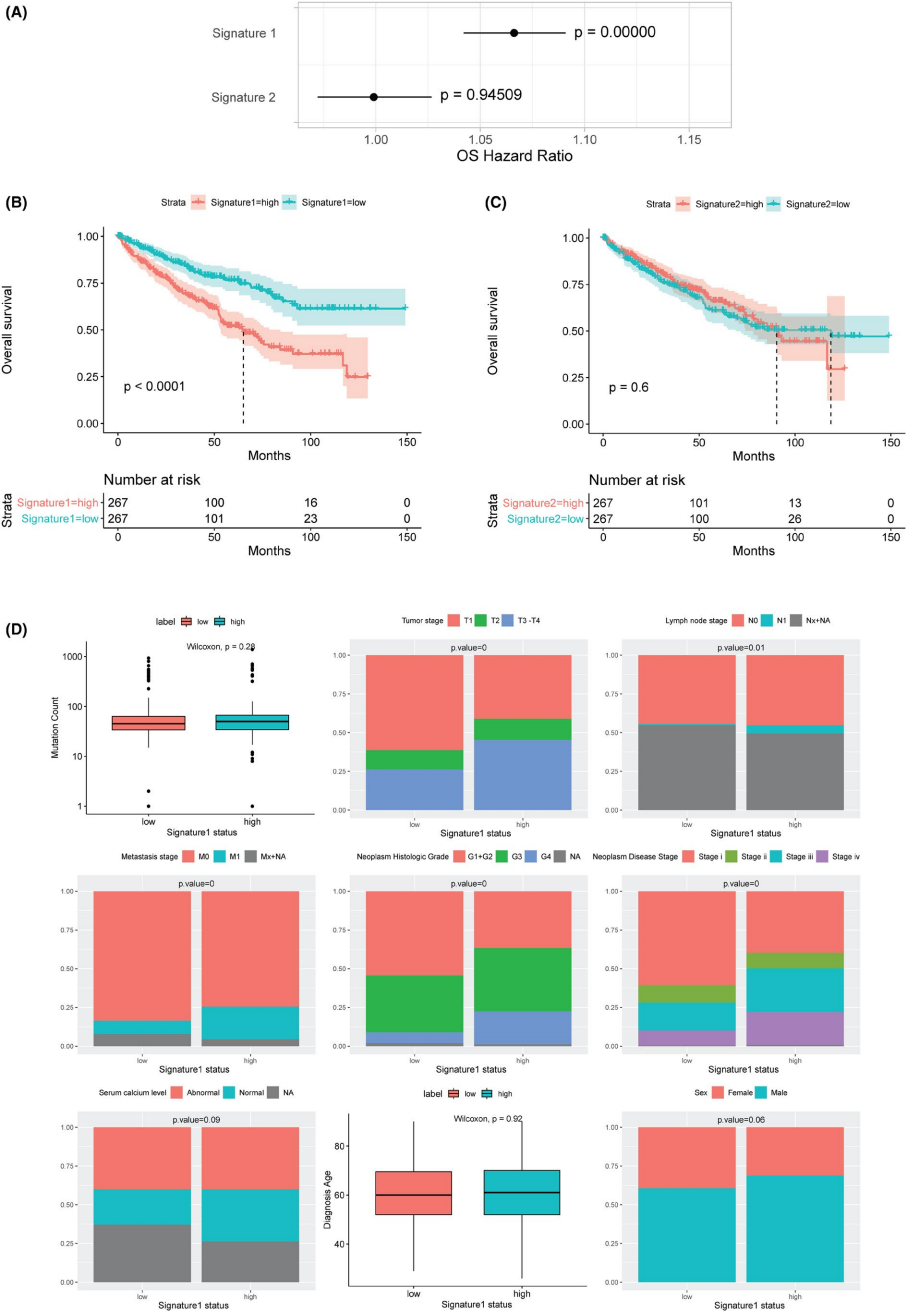
Overall Survival analysis of MUCIN signatures in ccRCC cohort. (A) Cox analysis of signature 1 and signature 2. (B) Survival analysis of patients with signature 1. (C) Survival analysis of patients with signature 2. (D) Clinical features of patients with low signature 1 or not

As clusters 1 and 2 were based on the expression data of the whole family of MUCIN members, which limited its application in clinical practice, we investigated whether simplified signatures may give sufficient performance. We exacted signature 1 (*MUC1*, *MUC12*, *MUC13*, *MUC16*, and *OVGP1*) from cluster 1 and signature 2 (*MUC4*, *MUC5B*, *MUC6*, *MUC20*, *EMCN*, and *MCAM*) from cluster 2, and each signature was weighted by hazard ratio (Figure 8A). Though signature 2 did not show a significant association with survival (*p* = 0.6), patients with signature 1 were associated with worse survival than those without the feature (*p *< 0.0001, Figure 8B,C). Then, we validated this predictor in the GSE29069 dataset, which was independent of the previous data. Although *MUC12* expression was absent and the sample size was limited in GSE29069, univariate Cox analysis indicated that each *MUCIN* in signature 1 (*MUC1*, *MUC13*, *MUC16*, *OVGP1*) were all associated with a trend with poor survival, especially *OVGP1* (Figure S3). The enrichment of signature 1 was associated with a trend of poor survival (*p* = 0.087, Figure S4). Interestingly, though there was no difference in the mutation counts and serum calcium level between samples with or without signature 1, a similar feature to cluster 1, including later tumor stage and disease stage, higher neoplasm histologic grade with more lymph node metastasis, and long‐distance metastasis, were also presented in samples with signature 1 (Figure 8D).

### 3.8 Tumor characteristics in signature 1

**FIGURE 9:**
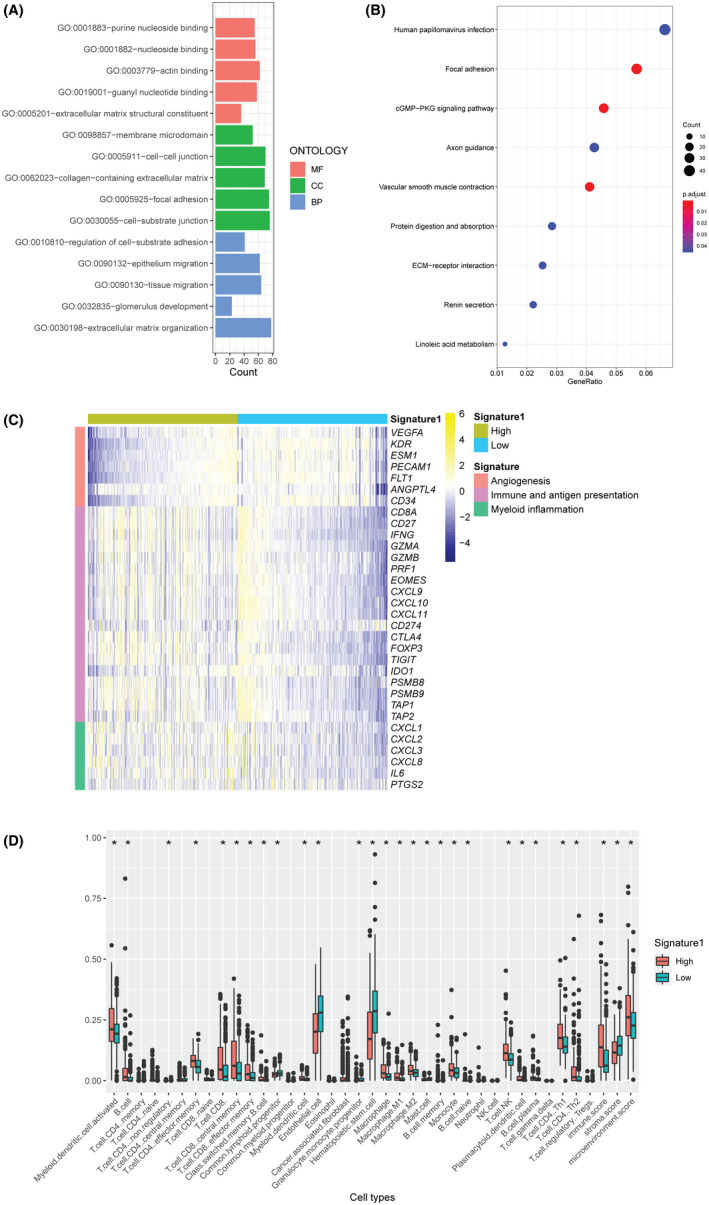
Signature 1 related immune biological pathways. (A) Gene ontology enrichment of DEGs. (B) KEGG enrichment of DEGs. (C) mRNA expressions heatmap of genes related to angiogenesis, immune and antigen presentation, and myeloid inflammation. (D) Different distributions of cells are estimated by xCell in low signature 1 status and high signature 1 status. DEG: differentially expressed genes; BP: biological process; CC: cellular component; MF: molecular function. * represent *p* < 0.05

The gene ontology analysis showed that DEGs in samples with signature 1 were mainly enriched for the following biological processes: cell‐substrate adhesion, epithelium migration, tissue migration, glomerulus development, and extracellular matrix organization (Figure 9A). The related signaling pathways were focal adhesion, cGMP−PKG signaling pathway, and ECM−receptor interaction (Figure 9B). Additionally, differential expression of genes involved in angiogenesis and immune and antigen presentation was observed in ccRCC samples with signature 1 (Figure 9C). A significantly higher level of the immune score but a lower level of stroma score was identified in samples with signature 1, with the increased presence of multiple types of infiltrated immune cells, such as activated myeloid dendritic cells, CD4 positive effector memory T cells, and CD8 positive T cells (Figure 9D).

## 4 DISCUSSION

*MUCIN*s, especially membrane‐bound *MUCIN*s, have been demonstrated to be associated with a shared tumor progression pattern,^12^ but the correlation of *MUCIN*s’ mutation and expression pattern and with ccRCC patients’ prognosis is not fully documented. Although various *MUCIN* members have been studied singly in ccRCC, the comprehensive and integrated role of *MUCIN*s in ccRCC patients’ prognosis is still unclarified.

In the present study, we analyzed the ccRCC data from the TCGA database to examine *MUCIN*s’ correlation with patients’ survival and identified cluster 1 expression pattern as being associated with poor survival. To simplify the prognostic biomarker, we successfully established a five‐*MUCIN* expression signature that significantly correlated with worse survival and validated in independent ccRCC patient datasets. This signature may potentially be used as a prognostic marker in further clinical practice.

The signature is composed of the overexpression of *MUC1*, *MUC12*, *MUC13*, *MUC16*, and *OVGP1*, which were all contribution variables associated with poor survival in ccRCC. The predictive power of the signature may lie in the function of each MUCIN gene, some but not all of which had been reported to have various functions in different types of cancer and/or kidney development. Among them, *MUC1* and *MUC16* were found to be associated with immune modulation and metastasis in cancer. The presence of *MUC16* neo‐antigen‐specific T cell clones and anti‐*MUC1* antibodies in cancer suggests that *MUCIN*s can serve as potential targets for developing cancer therapeutics.^38^ Notably, *MUCIN* mutations have been demonstrated to affect normal kidney function. For instance, *MUC1* mutations directly cause autosomal dominant tubulointerstitial kidney disease.^21^ *MUC1* could be directly regulated by HIF‐1‐alpha, whose aberrant expression has been found frequently in ccRCC.^22^, ^23^, ^24^ Moreover, *MUC1* can be involved in renal tumor development and can be considered as potential markers of ccRCC development and prognostic,^25^, ^26^ which provides a foundation for the development of a cancer vaccine (TG4010, based on *MUC1* and interleukin‐2) in the first‐line therapy of metastatic ccRCC patients.^27^ Previous studies also showed that aberrantly glycosylated MUC1 is overexpressed in most human epithelial cancers^28^ and it mediates enhanced expression of glucose uptake and metabolism genes, facilitating cancer cell survival and growth in multiple cancers.^29^, ^30^, ^31^ And importantly, MUC1 could induce resistance to anticancer drugs by upregulating the expression of multi‐drug resistance genes, for instance, multidrug resistance protein 1.^32^ MUC16 overexpression has also been linked to worse prognosis in intrahepatic cholangiocarcinoma and pancreatic tumors.^33^, ^34^ Otherwise, it was proposed as a suppressor in TLR‐mediated innate immune activation.^22^, ^35^ However, *MUC16* overexpression was not described in ccRCC before. Overexpression of *MUC12* and *MUC13* in ccRCC was found to promote RCC progression depending on c‐Jun/TGF‐β signaling, which was associated with poor prognosis by increasing RCC cell growth and cell invasion.^14^, ^16^ *OVGP1* was identified as an independent prognostic factor and had an association with drug resistance in ovarian cancer, and its elevation in serum could serve as an accuracy biomarker for ovarian cancer independent of CA125.^36^, ^37^ *OVGP1* overexpression had not been described in ccRCC before, either.

In addition to serving as a prognostic marker, the cluster 1 pattern and signature 1 may be considered for informing the selection of anti‐angiogenesis and/or immune checkpoint inhibitors. Nowadays, treatments targeting the aberrant vascular endothelial growth factor/receptor (VEGF/VEGFR) pathway and/or immune checkpoint inhibitors are among the main and most effective therapy for ccRCC. However, the determinants for therapy selection are still undefined. In the present analysis, cluster 1 and its related signature 1 revealed a higher level of microenvironment immune score and multiple infiltrated immune cells, including CD4 positive effector memory T cells, CD8 positive T cells, and macrophages. Meanwhile, a relatively lower enrichment in the angiogenesis signature was found in patients from cluster 1 and signature 1. Previous studies, including IMmotion 150, have identified the association between anti‐VEGFR therapy and angiogenesis signature, immune cell infiltration, and ICIs.^39^ *MUC1* had been found to engage with Siglec‐9, induced tumor‐associated macrophage‐like phenotype, and increased PD‐L1 expression in the tumor microenvironment.^40^ These results may suggest that ccRCC patients with signature 1 may benefit less from anti‐VEGFR monotherapy but have more response to ICIs and/or combination therapy instead.

However, our study also has distinct limitations. First, only 11 of 20 *MUCIN*s expression data were obtained for the prognosis‐related analysis, which might affect the outcome of the study. Besides, the sample size in the validation cohort was too small to fully validate the prognosis value of our established predictor. Though we found the potential difference in the sensitivity to anti‐VEGFR and/or immune checkpoint inhibitors between patients with or without signature 1 feature, the absence of direct evidence on the response to related therapy may need further study to evaluate its clinical application. Further research with local *MUCIN*s expression and immunotherapy data could better elucidate the relationship between our findings and the prognosis and management of patients with ccRCC.

In this study, we also found a higher prevalence of genetic alterations in *MUCIN*s in our cohort comparing with the TCGA database, but the genetic alterations in *MUCIN*s were not significantly correlated with ccRCC patients’ survival. The underlying reason for the higher mutation rate in our cohort is currently unclear, which may be related to the difference in the genetic background of Chinese and Western populations, or environmental exposure to different foods/medicines. The answer to this question awaits future investigation.

In conclusion, we successfully classified ccRCC patients into two clusters according to their expression patterns and deciphered each cluster's feature in the clinical and prognosis. Especially, a 5‐gene expression signature 1 extracted from cluster 1 could serve as a combined predictor marker and potentially help clinicians assign therapy choices for ccRCC patients.

## 5 DATA AND MATERIAL AVAILABILITY STATEMENT

The raw sequencing data files were deposited in the Chinese National Genomics Data Center(https://bigd.big.ac.cn/). Left biospecimens may be shared for academic research under prior approval from the government of China.

## CONFLICTS OF INTEREST

The authors have no conflict of interest to declare.

## AUTHOR’S CONTRIBUTION

HM: data collection, analysis, and drafting the article, XJ: manuscript revision, HH: data analysis, NS: data collection, CG: data collection, CY: data collection, GY: data collection, YW: design of this work and data analysis.

## ETHICAL STATEMENT

This study was approved by the ethics committee of Qilu Hospital and conducted under the principles of the Declaration of Helsinki and the Good Clinical Practice guidelines. All enrolled patients provided written informed consent.
